# Deep learning-based multiclass classification for the diagnosis of hand arthritis on digital radiography: a multicenter study

**DOI:** 10.3389/fmed.2026.1941638

**Published:** 2026-09-15

**Authors:** Chao Deng, Dan Yan, Cong Tang, Xuemei Deng, Chuanyu Li, Xikui Chen, Lihua Deng, Zhe Wu

**Affiliations:** 1 Department of Radiology, The First People’s Hospital of Neijiang, Neijiang, Sichuan, China; 2 Department of Radiology, Suining Central Hospital, Suining, Sichuan, China; 3 Department of Radiology, The Second People’s Hospital of Neijiang, Neijiang, Sichuan, China; 4 Department of Radiology, Neijiang Hospital of Traditional Chinese Medicine, Neijiang, Sichuan, China

**Keywords:** deep learning, gout, hand arthritis, osteoarthritis, radiography, rheumatoid arthritis

## Abstract

**Background:**

To develop and independently validate a deep learning framework for multiclass differentiation of osteoarthritis (OA), rheumatoid arthritis (RA), gouty arthritis (GA), and normal controls on hand digital radiographs.

**Methods:**

This retrospective multicenter study included 1,179 hand radiographs from 520 participants at four institutions. A three-center development cohort comprised 1,001 radiographs from 431 participants and was evaluated using patient-level stratified five-fold cross-validation. ResNet50, ResNet101, DenseNet121, and Vision Transformer (ViT) were trained using an identical pipeline. The internally best model, ViT, was retrained on the complete development cohort and evaluated once on an independent fourth-center cohort of 178 radiographs from 89 participants that was held out from training and model selection.

**Results:**

ViT achieved the numerically highest internal performance, with an accuracy of 0.937 ± 0.029 and a macro-*F*1 score of 0.937 ± 0.029. On independent external validation, ViT correctly classified 133 of 178 radiographs, with accuracy of 0.747, macro-precision of 0.756, macro-recall of 0.747, macro-specificity of 0.916, macro-*F*1 of 0.744, and macro-AUC of 0.917. External class-wise AUCs were 0.948 for RA, 0.873 for OA, 0.888 for GA, and 0.959 for normal controls.

**Conclusion:**

The transformer-based model showed strong internal performance and retained useful discriminative ability in an independent external cohort; however, the reduction in external accuracy and macro-*F*1 indicated limited cross-center transferability. These findings support the feasibility of the proposed approach while emphasizing the need for broader prospective validation and reader-performance studies before clinical deployment.

## 1 Introduction

Osteoarthritis (OA) is a leading cause of disability worldwide and represents a substantial and growing public health burden. According to the Global Burden of Disease 2023 study, OA accounts for a significant proportion of global years lived with disability, with prevalence increasing in parallel with population aging (1). In addition to OA, rheumatoid arthritis (RA) and gouty arthritis (GA) are major causes of hand arthritis. Although these conditions share similar clinical manifestations, including joint pain, stiffness, swelling, and functional impairment, their underlying pathophysiology, disease course, and therapeutic strategies differ substantially (2, 3). Accurate differentiation is therefore essential to guide appropriate management and prevent irreversible joint damage.

Medical imaging plays a pivotal role in OA management by enabling non-invasive visualization of joint structures for diagnosis, disease monitoring, and therapeutic evaluation (4). This is particularly relevant because the hand is a common site of OA involvement and hand OA can substantially affect daily function (5). Radiography (x-ray) remains the first-line imaging modality for hand arthritis due to its low cost, minimal radiation, rapid acquisition, and wide accessibility. However, it harbors three inherent limitations that compromise diagnostic reliability. First, compared to CT and MRI, radiography offers lower soft-tissue contrast and limited sensitivity for early inflammatory changes (6). Second, the two-dimensional projection of complex small hand joints can obscure subtle lesions due to anatomical superimposition (7). Third, and most critically, radiographic interpretation is highly subjective, leading to substantial inter- and intra-observer variability (8). This variability stems from both manual scoring methods and inconsistencies in image acquisition. Consequently, less experienced clinicians face considerable difficulty in accurately discriminating between arthritic subtypes, such as OA, RA, and GA, whose radiographic features often overlap, particularly in early disease stages (9). This diagnostic ambiguity contributes to misdiagnosis and delayed treatment. Deep learning offers potential solutions by automating feature detection and capturing subtle textural bone changes imperceptible to the human eye (6, 10, 11). However, current literature reveals two notable gaps. First, most OA research has focused on the knee using MRI, largely overlooking conventional radiography despite its accessibility advantages (12). Second, existing radiograph-based deep learning studies of hand arthritis have predominantly focused on RA detection or limited binary discrimination, with less attention to unified multiclass differentiation including OA and GA (12, 13).

Deep learning may assist radiographic interpretation by learning high-dimensional patterns of bone texture, joint alignment, and local structural change. Published work has demonstrated promising performance for RA detection and for differentiating RA from OA on hand radiographs (14, 15); recent studies have also addressed radiographic hand OA detection and grading (16). However, most available models remain disease-specific or binary/three-class, and fewer studies evaluate a unified multiclass framework that includes GA. Moreover, rigorous patient-level validation, transparent preprocessing, uncertainty reporting, and modern model comparison remain essential for medical-image AI studies.

Recent advances in medical image analysis have expanded beyond conventional convolutional neural networks. Vision Transformer (ViT) models use self-attention mechanisms to model long-range spatial relationships across image patches, which may be advantageous for hand radiographs where diagnostically relevant changes can be distributed across multiple small joints. Therefore, the present study compared ResNet50, ResNet101, DenseNet121, and ViT in a three-center development cohort using patient-level stratified five-fold cross-validation. The internally best-performing architecture was then retrained on the complete development cohort and tested on a fully held-out fourth-center cohort to assess independent external generalizability.

## 2 Materials and methods

This retrospective multicenter study was conducted in accordance with the Declaration of Helsinki. The requirement for informed consent was waived by the ethics committee because of the retrospective nature of the study.

### 2.1 Dataset construction

Hand digital radiographs were retrospectively collected from four tertiary medical centers in Sichuan Province, China: Neijiang First People's Hospital, Neijiang Second People's Hospital, Neijiang Hospital of Traditional Chinese Medicine, and Suining Central Hospital. Radiographs were acquired using DR systems from multiple vendors, including Philips Healthcare, GE Healthcare, and Shimadzu Corporation. A total of 598 patients or control participants who underwent hand DR imaging between September 2018 and June 2026 were screened. After excluding 43 participants without a definitive clinical diagnosis and 35 with non-standard positioning or image artifacts, 520 participants with 1,179 diagnostic-quality radiographs were included. Before model development, the cohort was separated by institution. The development cohort comprised 431 participants and 1,001 radiographs from Neijiang First People's Hospital, Neijiang Second People's Hospital, and Suining Central Hospital: RA (270 images), OA (261), GA (226), and normal controls (244). The independent external validation cohort comprised 89 participants and 178 radiographs from Neijiang Hospital of Traditional Chinese Medicine: RA (46 images), OA (50), GA (36), and normal controls (46). No participant or image from the external cohort was used during model training, cross-validation, architecture selection, hyperparameter selection, or threshold determination (Figure 1 and Table 1).

**Figure 1:**
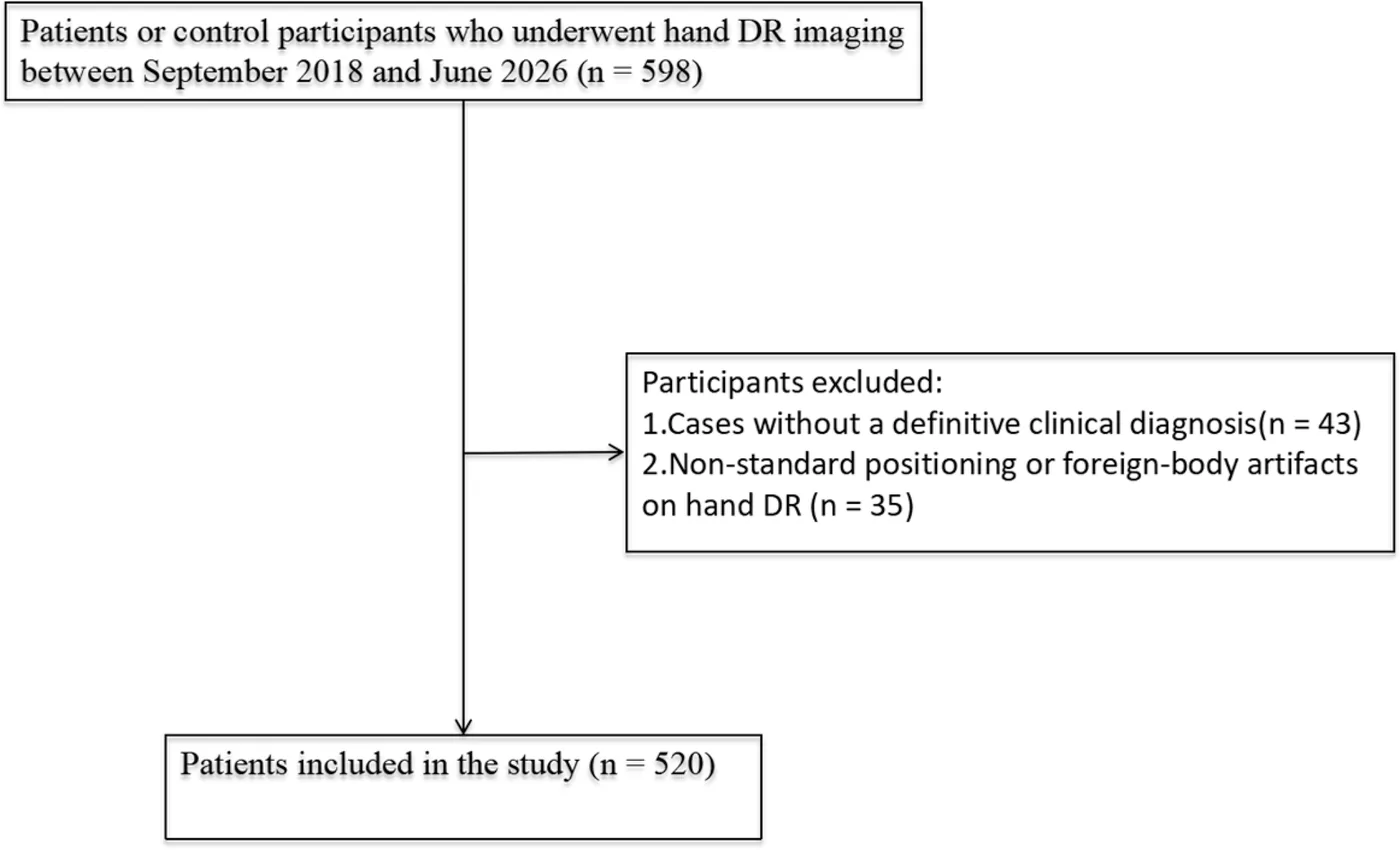
Participant selection flowchart.

**Table 1:** Baseline demographic characteristics of the development and independent external validation cohorts.

Variable	Development cohort (431 participants; 1,001 images)					External validation (89 participants; 178 images)				
	RA (*n* = 92)	OA (*n* = 113)	GA (*n* = 107)	Normal (*n* = 119)	*P* value	RA (*n* = 23)	OA (*n* = 25)	GA (*n* = 18)	Normal (*n* = 23)	*P* value
Age, years	60.11 ± 13.23	69.76 ± 13.03	63.36 ± 12.14	35.00 ± 11.59	<0.001	59.91 ± 12.40	67.40 ± 10.53	54.94 ± 15.02	38.00 ± 10.04	<0.001
Sex, *n* (%)					<0.001					<0.001
Male	11 (11.96%)	32 (28.32%)	87 (81.31%)	63 (52.94%)		5 (21.74%)	11 (44.00%)	16 (88.89%)	12 (52.17%)	
Female	81 (88.04%)	81 (71.68%)	20 (18.69%)	56 (47.06%)		18 (78.26%)	14 (56.00%)	2 (11.11%)	11 (47.83%)	
Images, n	270	261	226	244		46	50	36	46	

Values are mean ± standard deviation or *n* (%). *P* values compare the four diagnostic groups within each cohort (one-way analysis of variance for age and chi-square test for sex). Participant counts are shown in the column headings; image counts are shown in the final row.

### 2.2 Research workflow

The overall research workflow is presented in Figure 2. The study followed a structured deep learning framework consisting of data preparation, image preprocessing, model development, cross-validation, and diagnostic prediction. Initially, hand radiographs were collected and categorized into four diagnostic groups: RA, OA, GA, and normal controls. All images then underwent standardized preprocessing, including resizing to a unified input resolution and intensity normalization. Data augmentation was applied only to the training subset within each cross-validation fold. Patient-level stratified five-fold cross-validation was used for model development and evaluation. These procedures were implemented to ensure input consistency, reduce patient-level leakage, and improve the reliability of internal performance estimation. Model development was performed using a transfer learning strategy based on four deep learning architectures: ResNet50, ResNet101, DenseNet121, and ViT. For the CNN-based models, the original ImageNet classification layer was replaced with a four-node fully connected output layer corresponding to the predefined diagnostic categories. For ViT, the final classification head was similarly replaced with a four-node output layer. The trained models generated probability scores for each class, and the category with the highest Softmax probability was defined as the final diagnostic output.

**Figure 2:**
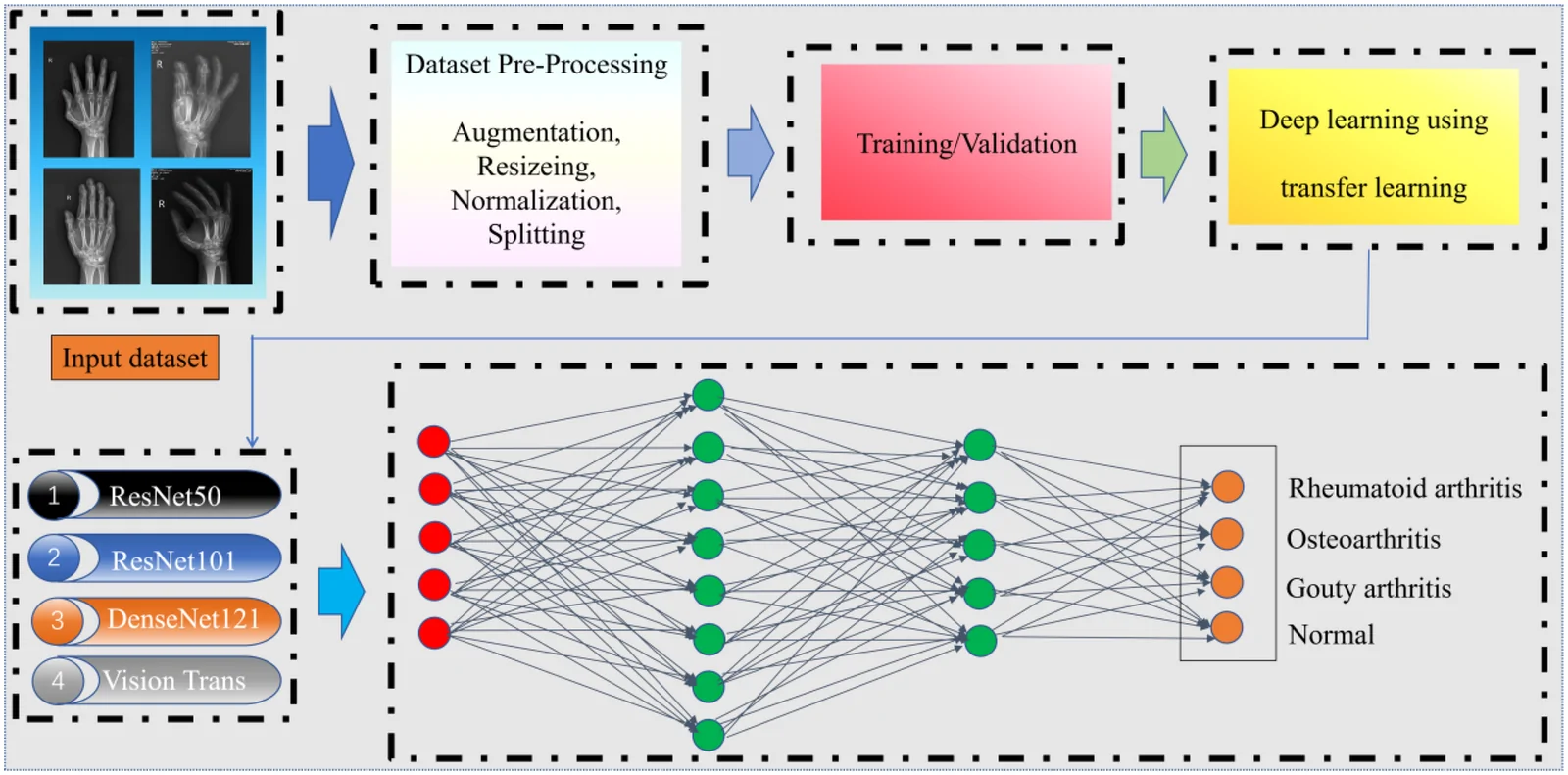
Overall workflow of the deep learning framework.

### 2.3 Image annotation and preprocessing

Ground-truth labels were assigned according to composite clinical-radiographic diagnoses rather than radiographic appearance alone. Final diagnoses of RA, GA, and OA were based on established clinical records and corresponding radiographic features, whereas normal controls had no clinical or radiographic evidence of hand arthritis. Each image was independently reviewed by two board-certified musculoskeletal radiologists, and disagreements were adjudicated by a third senior musculoskeletal radiologist. The annotation protocol included diagnostic category, projection view, and image-quality assessment. The original paired pre-adjudication ratings were not retained in a structured dataset; therefore, a retrospective Cohen's kappa coefficient could not be calculated reliably, and the adjudicated consensus label was used as the reference standard.

All radiographs were converted to grayscale images, resized to 512 × 512 pixels, and normalized before model input. Data splitting within the development cohort was performed at the participant level, ensuring that images from the same participant were never assigned to different folds. During training, augmentation was restricted to the training subset and included random horizontal flipping, limited-angle random rotation, random resized cropping, and mild brightness/contrast adjustment. The independent external cohort underwent the same deterministic resizing and normalization but no augmentation. Age and sex distributions for both cohorts are summarized in Table 1.

### 2.4 Patient-level five-fold cross-validation

Patient-level stratified five-fold cross-validation was used exclusively for model development and internal evaluation in the three-center development cohort. All images belonging to the same participant were assigned to the same fold to prevent leakage from paired or multi-view radiographs. Stratification was performed according to diagnostic category. In each fold, four-fifths of participants were used for training and one-fifth for validation. Internal performance was reported as mean ± standard deviation across the five folds, together with pooled confusion-matrix summaries.

### 2.5 Independent external validation

After completion of the internal model comparison, ViT was selected because it achieved the numerically highest cross-validated accuracy and macro-*F*1 score. The model was retrained using all 1,001 radiographs in the development cohort with the prespecified preprocessing procedures and hyperparameters. The 178 radiographs from Neijiang Hospital of Traditional Chinese Medicine remained completely unavailable during model development and were evaluated once without threshold adjustment or model recalibration. External predictions were generated by selecting the class with the highest softmax probability.

### 2.6 Model architectures

Four deep learning architectures were evaluated under the same preprocessing, training, and evaluation pipeline: ResNet50, ResNet101, DenseNet121, and ViT. ResNet50 and ResNet101 were selected to evaluate the influence of residual network depth on radiographic feature extraction. DenseNet121 was included because dense feature reuse may facilitate gradient propagation and preserve subtle structural information in small joints. ViT was added as a transformer-based comparator to assess whether global self-attention could improve multiclass differentiation of arthritis subtypes on hand radiographs.

For the CNN-based models, the original ImageNet classification layer was replaced with a fully connected layer containing four output nodes corresponding to OA, RA, GA, and normal controls. For ViT, the final classification head was similarly replaced with a four-class output layer. The predicted class was determined according to the maximum Softmax probability.

#### 2.6.1 ResNet50 classification network

ResNet50 is a deep residual convolutional neural network that uses bottleneck residual blocks and identity shortcut connections to facilitate gradient propagation and alleviate performance degradation during deep network training. The input image is first processed by an initial 7 × 7 convolutional layer followed by batch normalization, ReLU activation, and a stride-2 max-pooling layer, resulting in early spatial downsampling. The network then passes through four residual stages containing 3, 4, 6, and 3 bottleneck blocks, respectively. Each bottleneck block consists of 1 × 1, 3 × 3, and 1 × 1 convolutional layers, which reduce computational complexity while preserving representational capacity. Finally, global average pooling is applied, and the original classification layer is replaced with a task-specific fully connected layer with four output nodes corresponding to the predefined diagnostic categories (17).

#### 2.6.2 ResNet101 classification network

ResNet101 is a deep residual network that employs bottleneck residual modules and identity shortcut connections. The input image is processed by an initial 7 × 7 convolutional layer followed by a stride-2 max-pooling layer, resulting in a reduction of the spatial resolution to one-quarter of the original size. The network then passes through four residual stages consisting of 3, 4, 23, and 3 bottleneck modules, respectively, followed by global average pooling and a task-specific fully connected layer with four output nodes (17).

#### 2.6.3 DenseNet121 classification network

DenseNet121 is composed of densely connected convolutional blocks in which each layer receives the feature maps of all preceding layers within the same dense block (feature concatenation). The standard DenseNet121 architecture contains four dense blocks with 6, 12, 24, and 16 convolutional layers, interleaved with transition layers for feature compression and spatial downsampling. A global average pooling layer is applied before the final four-node fully connected classifier (18).

#### 2.6.4 Vision transformer classification network

ViT is a transformer-based image classification architecture that applies the self-attention mechanism directly to image patches rather than relying on convolutional operations. In ViT, the input image is divided into fixed-size patches, and each patch is linearly embedded into a token representation. A learnable classification token and positional embeddings are then added to preserve global image-level representation and spatial information. These token sequences are processed by stacked transformer encoder layers composed of multi-head self-attention and feed-forward networks, enabling the model to capture long-range spatial dependencies across the entire radiograph. In this study, the original classification head was replaced with a fully connected layer containing four output nodes, followed by a Softmax activation function for multiclass prediction (19).

### 2.7 Training details and hyperparameter settings

All models were implemented using PyTorch 2.1.0 and Python 3.8.0 and trained on a workstation equipped with an NVIDIA A100 GPU and 32 GB RAM. Model training used stochastic gradient descent with momentum, an initial learning rate of 0.001, momentum of 0.9, weight decay of 1 × 10^−4^, batch size of 8, and 100 epochs. A cosine-annealing learning-rate schedule was applied with a minimum learning rate of 1 × 10^−6^. The same preprocessing, augmentation strategy, training schedule, and hyperparameters were applied across architectures. For external validation, the selected ViT model was retrained on the complete development cohort using the same prespecified settings; the external cohort was used only for the final locked evaluation.

Because this study involved a four-class classification task, categorical cross-entropy loss was used, as defined in Equation 1:

$$L=-\frac{1}{N}\sum_{i=1}^{N}\sum_{c=1}^{C}\mathrm{yic}\log\;(\mathrm{pic})$$ (1)

Where *N* denotes the number of training samples, *C* denotes the number of diagnostic categories, _yic_ is the one-hot encoded ground-truth label for class *c*, and _pic_ is the predicted Softmax probability for class *c*.

### 2.8 Model evaluation

Model performance was evaluated using accuracy, precision, recall, specificity, *F*1 score, confusion matrix analysis, and receiver operating characteristic curve analysis. For multiclass evaluation, each diagnostic category was assessed using a one-vs.-rest strategy. Class-wise AUCs and macro-averaged metrics were calculated. Internal performance was summarized across folds, whereas external metrics were calculated once from the locked 178-image cohort. All classification metrics were derived from true positives, true negatives, false positives, and false negatives, as defined in Equations 2–6:

$$\mathrm{Accuracy}=\frac{\mathrm{TP}+\mathrm{TN}}{\mathrm{TP}+\mathrm{TN}+\mathrm{FP}+\mathrm{FN}}\;\times 100\text{\%}$$ (2)

$$\mathrm{Recall}=\frac{\mathrm{TP}}{\mathrm{TP}+\mathrm{FN}}\;\times 100\text{\%}$$ (3)

$$\mathrm{Specificity}=\frac{\mathrm{TN}}{\mathrm{TN}+\mathrm{FP}}\;\times 100\text{\%}$$ (4)

$$\mathrm{Precision}=\frac{\mathrm{TP}}{\mathrm{TP}+\mathrm{FP}}\;\times 100\text{\%}$$ (5)

$$F1\text{-}\mathrm{Score}=2*\frac{\mathrm{Precision}\;\;*\;\;\mathrm{Recall}}{\mathrm{Precision}+\mathrm{Recall}}\;\times 100\text{\%}$$ (6)

### 2.9 Statistical analysis

Internal precision, recall, specificity, *F*1 score, and accuracy were summarized as the mean ± standard deviation across the five folds. Class-wise AUCs were calculated from the mean ROC curves across the five folds. External accuracy and class-wise and macro-averaged precision, recall, specificity, *F*1 score, and AUC were calculated once from the independent external validation cohort and are therefore reported as point estimates. Macro averages were unweighted across classes. Age was compared across diagnostic groups using one-way analysis of variance, and sex distributions were compared using the chi-square test within each cohort. Because prediction-level outputs for all internal folds were not available for paired testing, between-model differences were interpreted descriptively rather than as evidence of statistical superiority.

## 3 Results

### 3.1 Overall model performance

The cross-validated performance of the four models is summarized in Tables 2, 3. ViT showed the numerically highest overall internal performance, with an accuracy of 0.937 ± 0.029, macro-precision of 0.939 ± 0.029, macro-recall of 0.937 ± 0.030, macro-specificity of 0.979 ± 0.010, and macro-*F*1 score of 0.937 ± 0.029. Based on the pooled five-fold validation confusion matrix, ViT correctly classified 938 of 1,001 validation images, corresponding to a pooled accuracy of 0.937. ResNet50 and ResNet101 each correctly classified 927 of 1,001 images, with overall accuracies of 0.926 ± 0.015 and 0.926 ± 0.013, respectively. DenseNet121 correctly classified 926 of 1,001 images, with an accuracy of 0.925 ± 0.011. These findings suggest that ViT provided the most favorable overall performance in this multiclass classification task, although the differences among models were modest. The cross-validation results for each model are shown in Figure 3 (DenseNet121), 4 (ResNet50), 5 (ResNet101), and 6 (ViT).

**Figure 3:**
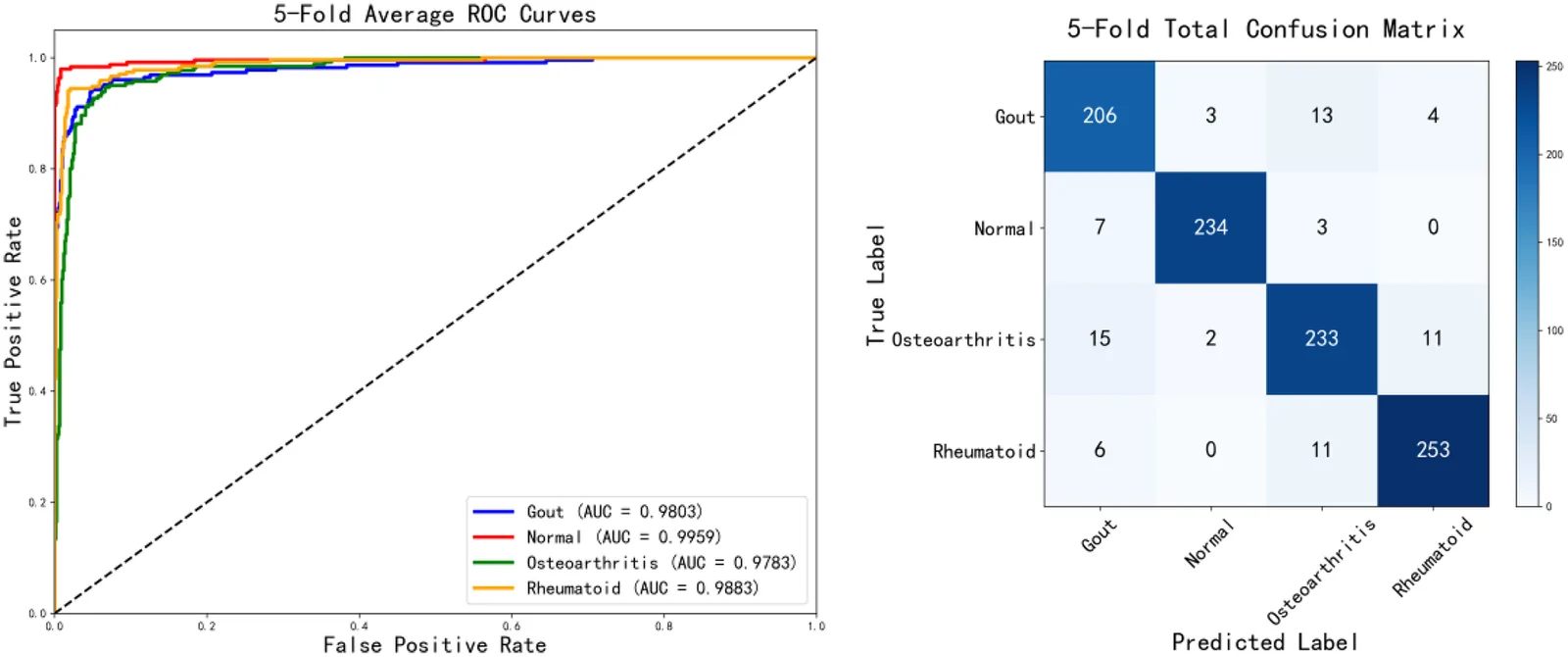
Cross-validation results of the DenseNet121-based classification model.

**Figure 4:**
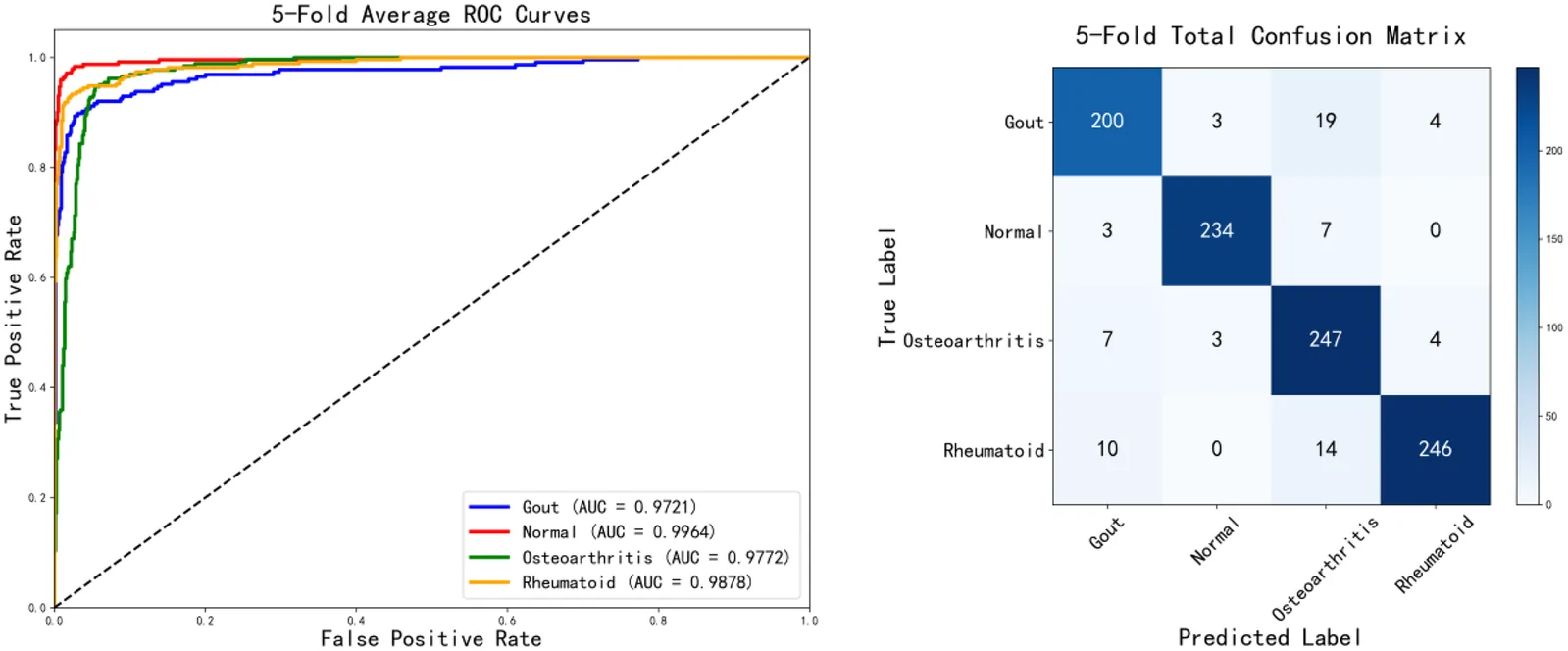
Cross-validation results of the ResNet50-based classification model.

**Figure 5:**
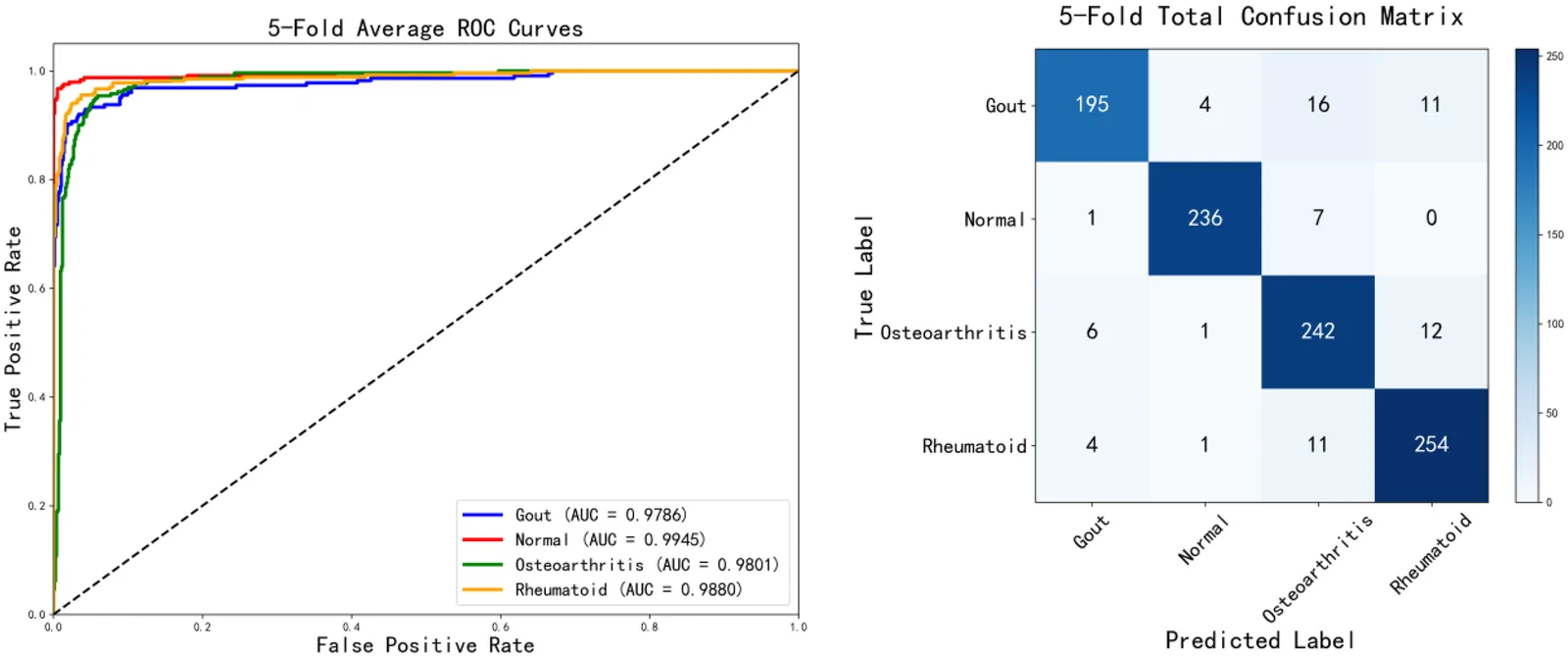
Cross-validation results of the ResNet101-based classification model.

**Figure 6:**
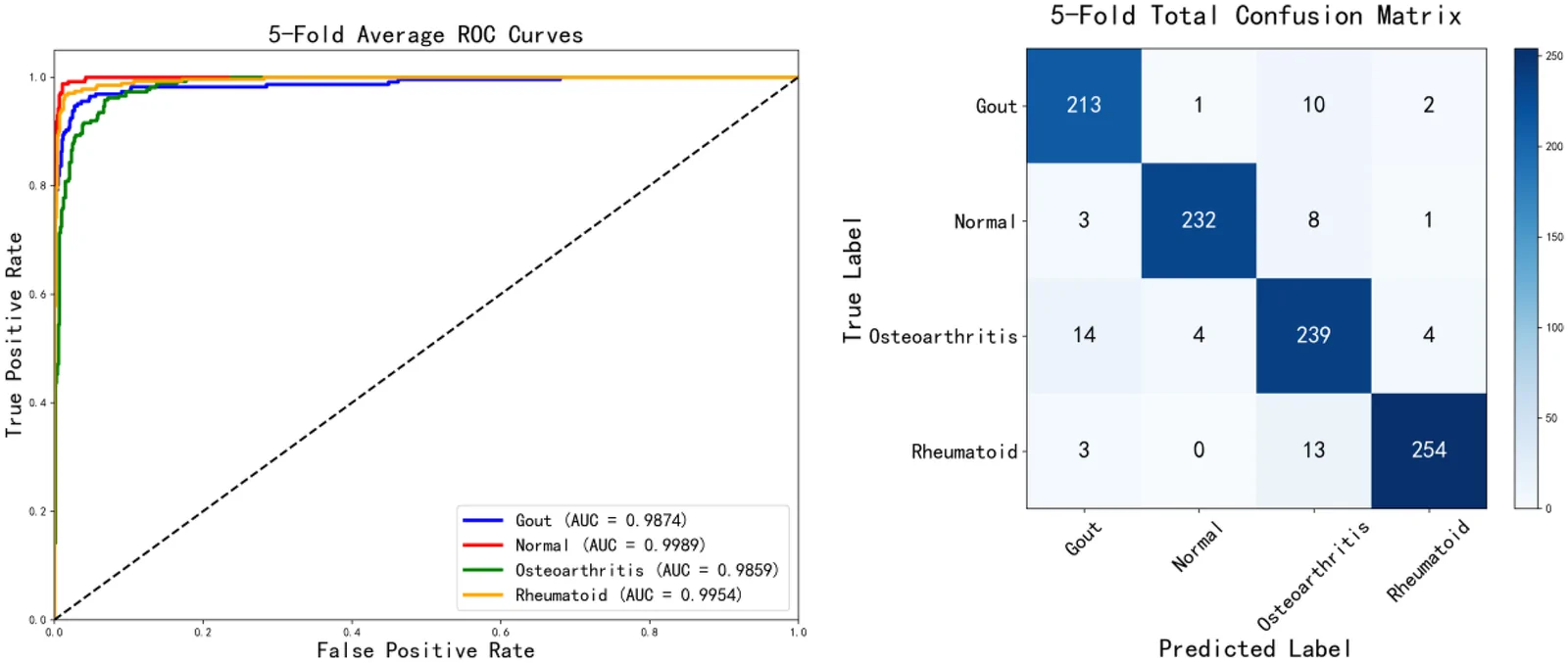
Cross-validation results of the ViT-based classification model.

**Table 2:** Class-wise internal cross-validation performance of each deep learning model.

Methods	Category	AUC	Precision	Recall	Specificity	*F*1 score
ResNet50	Rheumatoid arthritis	0.988	0.970 ± 0.030	0.911 ± 0.036	0.989 ± 0.011	0.939 ± 0.019
	Osteoarthritis	0.977	0.863 ± 0.041	0.947 ± 0.047	0.946 ± 0.019	0.902 ± 0.026
	Gouty arthritis	0.972	0.914 ± 0.048	0.885 ± 0.076	0.974 ± 0.016	0.896 ± 0.031
	Normal controls	0.996	0.975 ± 0.024	0.959 ± 0.022	0.992 ± 0.007	0.967 ± 0.020
ViT	Rheumatoid arthritis	0.995	0.974 ± 0.023	0.941 ± 0.054	0.990 ± 0.008	0.956 ± 0.030
	Osteoarthritis	0.986	0.889 ± 0.063	0.916 ± 0.036	0.958 ± 0.025	0.901 ± 0.041
	Gouty arthritis	0.987	0.913 ± 0.028	0.942 ± 0.065	0.974 ± 0.007	0.927 ± 0.045
	Normal controls	0.999	0.980 ± 0.025	0.951 ± 0.034	0.993 ± 0.008	0.964 ± 0.020
ResNet101	Rheumatoid arthritis	0.988	0.918 ± 0.028	0.941 ± 0.022	0.969 ± 0.012	0.929 ± 0.013
	Osteoarthritis	0.980	0.877 ± 0.024	0.927 ± 0.015	0.954 ± 0.010	0.901 ± 0.018
	Gouty arthritis	0.979	0.946 ± 0.018	0.863 ± 0.052	0.986 ± 0.005	0.902 ± 0.033
	Normal controls	0.995	0.976 ± 0.029	0.967 ± 0.028	0.992 ± 0.010	0.971 ± 0.027
DenseNet121	Rheumatoid arthritis	0.988	0.946 ± 0.038	0.937 ± 0.025	0.980 ± 0.016	0.941 ± 0.018
	Osteoarthritis	0.978	0.897 ± 0.028	0.893 ± 0.020	0.964 ± 0.011	0.900 ± 0.010
	Gouty arthritis	0.980	0.885 ± 0.071	0.911 ± 0.032	0.964 ± 0.023	0.897 ± 0.043
	Normal controls	0.996	0.980 ± 0.022	0.959 ± 0.026	0.993 ± 0.007	0.969 ± 0.015

AUC values were derived from the mean ROC curves across the five folds; all other metrics are presented as the mean ± standard deviation across folds.

**Table 3:** Overall internal cross-validation performance of each deep learning model.

Methods	Macro-precision	Macro-recall	Macro-specificity	Macro-*F*1 score	Accuracy
ResNet50	0.931 ± 0.014	0.925 ± 0.017	0.975 ± 0.005	0.926 ± 0.016	0.926 ± 0.015
ViT	0.939 ± 0.029	0.937 ± 0.030	0.979 ± 0.010	0.937 ± 0.029	0.937 ± 0.029
ResNet101	0.929 ± 0.011	0.925 ± 0.013	0.975 ± 0.004	0.926 ± 0.013	0.926 ± 0.013
DenseNet121	0.927 ± 0.012	0.925 ± 0.012	0.975 ± 0.004	0.925 ± 0.012	0.925 ± 0.011

### 3.2 Class-wise diagnostic performance

At the class level, ViT achieved internal AUCs of 0.995 for RA, 0.986 for OA, 0.987 for GA, and 0.999 for normal controls. Based on the pooled five-fold cross-validation confusion matrix, ViT correctly classified 254 of 270 RA images, 239 of 261 OA images, 213 of 226 GA images, and 232 of 244 normal-control images, corresponding to recalls of 0.941, 0.916, 0.942, and 0.951, respectively. ViT achieved the highest AUC for OA among the four evaluated models, while the corresponding *F*1 scores were similar, ranging from 0.900 to 0.902. All models also demonstrated high discrimination for normal controls, with AUCs ranging from 0.995 to 0.999.

### 3.3 Independent external validation performance

On the independent external validation cohort (178 radiographs), the selected ViT model correctly classified 133 images, achieving an overall accuracy of 0.747. The macro-precision, macro-recall, macro-specificity, macro-*F*1, and macro-AUC were 0.756, 0.747, 0.916, 0.744, and 0.917, respectively (Table 4). Class-wise performance (Figure 7) revealed AUCs of 0.948 for RA, 0.888 for GA, 0.873 for OA, and 0.959 for normal controls. The confusion matrix indicated that the model correctly identified 29/46 RA, 39/50 OA, 27/36 GA, and 38/46 normal images. RA exhibited high precision (0.879) but low recall (0.630), suggesting a tendency toward under-detection. GA achieved a recall of 0.750 but a lower precision of 0.614, reflecting a higher rate of false-positive predictions. OA and normal controls yielded *F*1 scores of 0.750 and 0.817, respectively.

**Table 4:** Independent external validation performance of the selected vision transformer model.

Category	AUC	Precision	Recall	Specificity	*F*1 score	Accuracy
Rheumatoid arthritis	0.948	0.879	0.630	0.970	0.734	
Osteoarthritis	0.873	0.722	0.780	0.883	0.750	
Gouty arthritis	0.888	0.614	0.750	0.880	0.675	
Normal controls	0.959	0.809	0.826	0.932	0.817	
Macro average	0.917	0.756	0.747	0.916	0.744	
Overall						0.747

**Figure 7:**
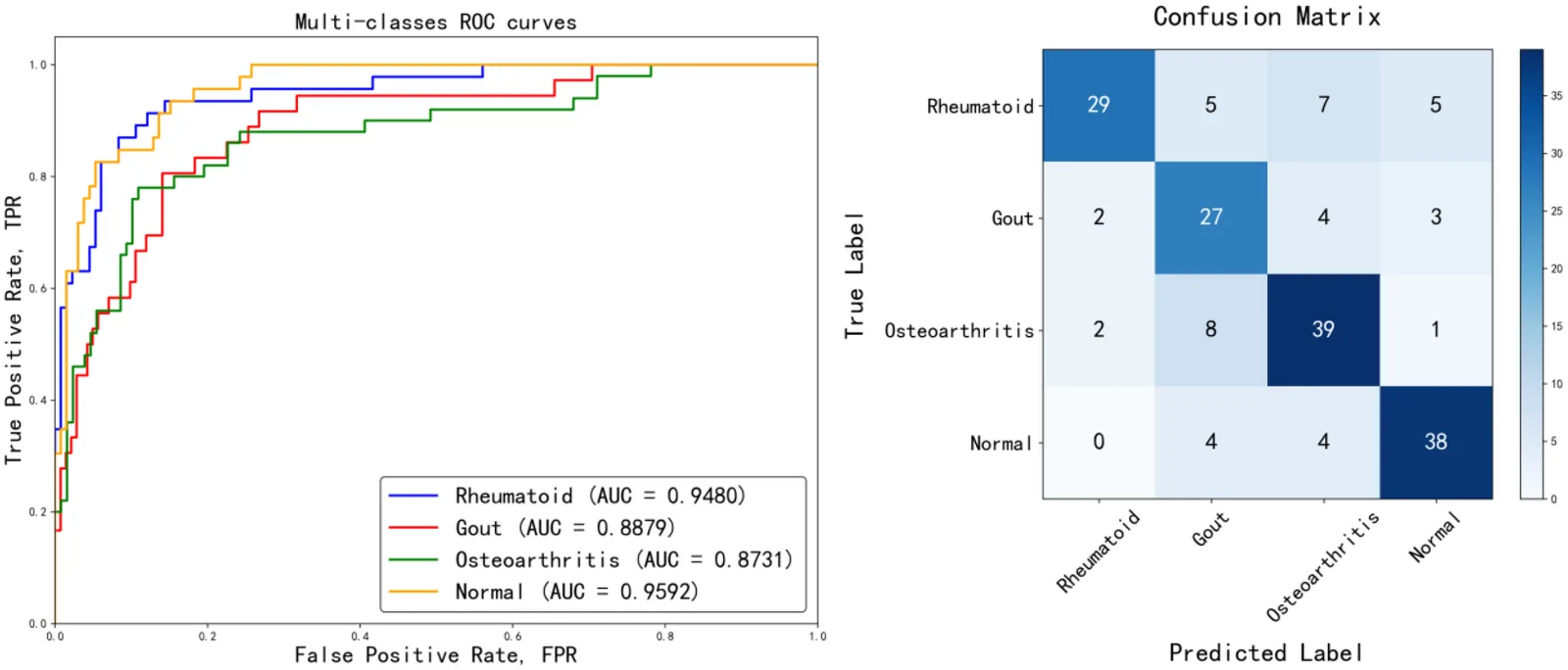
Independent external validation results of the ViT-based model.

### 3.4 Model interpretability

Model interpretability was assessed using class activation mapping (CAM), as shown in Figure 8. Representative RA, GA, and OA cases showed activation near radiographically relevant joint regions, providing qualitative support for the plausibility of the learned imaging features. Because heatmap-based explanations are qualitative, they were interpreted as supportive visualization rather than definitive evidence of causal model reasoning.

**Figure 8:**
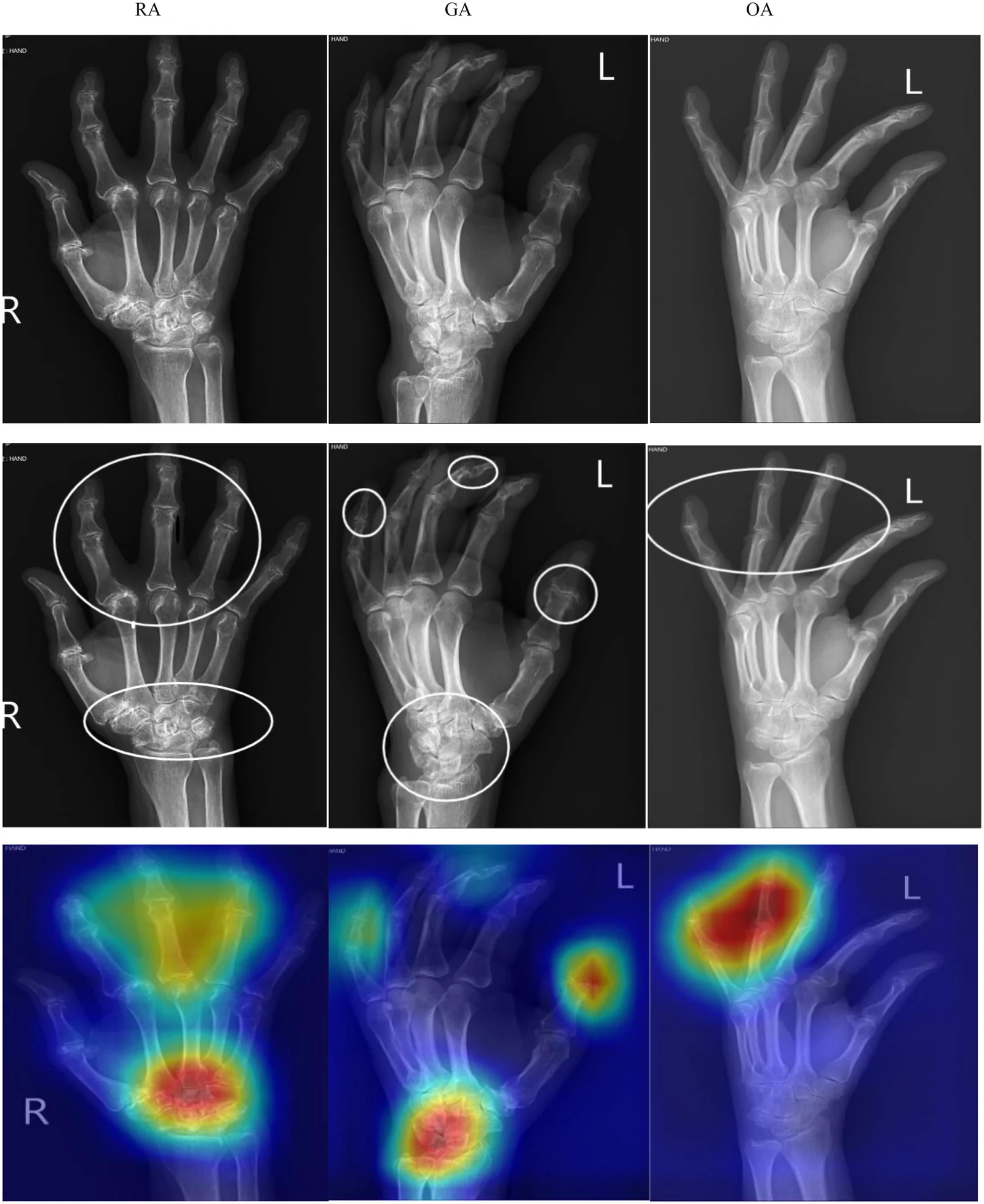
Representative radiographs and model-interpretability visualizations for rheumatoid arthritis, gouty arthritis, and osteoarthritis. The top row shows the original radiographs, the middle row shows highlighted radiographic abnormalities, and the bottom row shows the corresponding CAM heatmaps.

## 4 Discussion

AI-assisted medical imaging has demonstrated increasing value in OA research, contributing to enhanced diagnostic accuracy, quantitative disease assessment, and prognostic modeling (20, 21). In this multicenter study, we developed a four-class deep learning framework in three centers and independently evaluated the selected model in a fourth center. ViT showed the numerically best internal performance, which may relate to its ability to integrate distributed abnormalities across multiple small joints through global self-attention. Importantly, the independent external test provided a more stringent estimate of generalizability than pooled multicenter cross-validation alone.

Although ViT showed the best internal performance, differences among ViT, ResNet50, ResNet101, and DenseNet121 were modest. ResNet50 and ResNet101 achieved similar accuracies, suggesting that greater residual depth did not materially improve performance in the development cohort. DenseNet121 also remained competitive. Accordingly, the internal comparison justified selecting ViT for external testing but did not establish definitive architectural superiority.

External performance was substantially lower than internal performance: accuracy decreased from 0.937 to 0.747, and macro-*F*1 decreased from 0.937 to 0.744. Nevertheless, class-wise AUCs remained between 0.873 and 0.959, suggesting that discriminatory ranking was partly preserved while fixed multiclass decision boundaries were less transferable. The error pattern was clinically relevant: RA had relatively high precision but low recall, whereas GA had acceptable recall but lower precision. This gap may reflect differences in scanner vendors, acquisition protocols, case mix, demographic composition, and disease severity between centers; the present data cannot isolate the contribution of each factor. The findings therefore demonstrate partial external generalizability rather than deployment-ready performance.

From a clinical perspective, radiography is the most accessible first-line imaging modality for hand arthritis evaluation. A robust radiograph-based classifier may therefore serve as a screening decision-support tool, helping reduce variability and prioritize cases for specialist review. However, many existing artificial intelligence (AI) studies predominantly focus on advanced imaging modalities, such as CT and MRI. In contrast, conventional radiography remains comparatively underutilized, despite its broad accessibility and well-established clinical role in musculoskeletal assessment (9, 22, 23). Current AI research in OA has largely concentrated on major weight-bearing joints, particularly the knee, with substantially fewer studies addressing the hip and only limited investigations focusing on the hand and foot joints (24). Moreover, AI-assisted analyses of hand radiographs have primarily focused on the detection and grading of RA. In contrast, research aimed at differentiating multiple arthritis subtypes, including RA, OA, and GA, remains limited, with studies specifically targeting gout being particularly scarce (25). Üreten et al. reported encouraging results in distinguishing RA, OA, and normal hand radiographs using a combined YOLOv4 and VGG-16 framework (14). Nevertheless, the diagnosis of GA presents distinct challenges, partly due to its underrepresentation in radiographic datasets and the limited availability of annotated hand radiographs. Such data imbalance may compromise model generalization and increase the risk of overfitting. Furthermore, hand arthritis often manifests with complex and heterogeneous radiographic features-including joint deformities, marginal erosions, joint space narrowing, and tophaceous deposits-which are critical for accurate diagnosis yet may be subtle and subject to observer variability during manual radiographic interpretation. The diagnostic performance observed in the present study is comparable to previously reported AI systems developed for hand arthritis on radiography. Üreten and Maraş reported 80.6% accuracy for three-class discrimination and AUC values up to 0.96–0.97 in binary settings (14). Ma et al. achieved 87.2% accuracy with AUCs of 0.975 for arthritis detection and 0.955 for RA discrimination (15).

In comparison with previous radiograph-based studies of hand arthritis, the present study achieved strong multiclass diagnostic performance within a unified four-category framework. Üreten et al. reported encouraging results in distinguishing RA, OA, and normal hand radiographs using a combined YOLOv4 and VGG-16 framework (14), and Ma et al. demonstrated the feasibility of deep learning for discriminating RA from OA on hand radiographs (15). For radiographic hand osteoarthritis, prior work has also shown that automated machine-learning approaches can support disease detection and grading (16). Unlike these previous studies, the present work incorporated RA, OA, GA, and normal controls within a single multiclass framework and evaluated both convolutional and transformer-based architectures using patient-level stratified five-fold cross-validation.

Among the four evaluated architectures, ViT achieved the numerically highest overall internal performance, with an accuracy of 0.937 ± 0.029 and a macro-*F*1 score of 0.937 ± 0.029. This finding suggests that global self-attention may provide complementary advantages for hand radiographs, where diagnostically relevant abnormalities are often distributed across multiple small joints. ResNet50, ResNet101, and DenseNet121 also showed robust and comparable performance, indicating that convolutional feature extraction remains effective for small-joint radiographic classification. However, the performance differences among models were modest; therefore, the present findings should be interpreted as evidence of promising model performance rather than definitive superiority of a single architecture.

Despite their strong predictive performance, deep learning models are frequently criticized as “black boxes” because of limited interpretability, which constrains clinical adoption in medical image analysis (26, 27). To address this limitation, explainable artificial intelligence (XAI) techniques, including CAM, have been increasingly used to identify image regions driving model predictions (28, 29). These approaches enhance transparency and support integration of AI systems into diagnostic workflows. Explainability is essential in high-stakes medical applications, where clinicians require insight into model reasoning to ensure trust and patient safety. Systematic reviews have shown that XAI methods improve interpretability by highlighting salient regions and feature contributions, thereby strengthening clinician confidence and facilitating regulatory acceptance (30, 31). In the present study, activation heatmaps generally highlighted regions around clinically relevant joints, providing qualitative support for the plausibility of the learned imaging features. Therefore, CAM findings should be regarded as qualitative supportive evidence for model plausibility rather than definitive proof of clinically causal reasoning.

Several strengths of this study should be noted. First, the study used a true center-level split: three institutions formed the development cohort and a fourth institution was completely held out for independent external validation. Second, the four-class framework reflects a clinically relevant differential diagnosis by including RA, OA, GA, and normal controls. Third, patient-level stratified cross-validation prevented leakage from paired or multi-view radiographs in the development cohort. Fourth, the baseline demographic table makes age and sex imbalances transparent. Finally, CAM was used as qualitative supportive evidence for model plausibility, while the interpretation was appropriately limited.

## 5 Limitations

Several limitations should be acknowledged. First, although an independent external cohort was included, it was relatively small and derived from a single hospital within the same province; validation in larger, geographically diverse, and prospective cohorts is therefore warranted. Second, differences in age and sex among the diagnostic groups may have introduced demographic confounding, and matched sensitivity analyses were not available. Third, inter-rater reliability could not be quantified because the original independent reader-level assessments before adjudication were not retained. Finally, the analysis was performed at the individual-image level without participant-level fusion of radiographs from different views, and relevant clinical variables were not incorporated into the model.

## 6 Conclusions

In conclusion, this multicenter retrospective study developed a deep learning framework for four-class differentiation of hand arthritis and evaluated it using both patient-level internal cross-validation and a completely held-out external center. ViT achieved the numerically highest internal performance and retained good class-wise discrimination externally, but external accuracy and macro-*F*1 were 0.747 and 0.744, respectively. The model should therefore be regarded as a research-stage decision-support approach rather than a clinically validated diagnostic system. Larger multicenter prospective validation, preserved reader-level annotations, demographic sensitivity analyses, multi-view fusion, and reader-performance studies are required before clinical translation.

## Data Availability

The original contributions presented in the study are included in the article/Supplementary Material, further inquiries can be directed to the corresponding authors.

## References

[1] GBD 2023 Disease and Injury and Risk Factor Collaborators. Burden of 375 diseases and injuries, risk-attributable burden of 88 risk factors, and healthy life expectancy in 204 countries and territories, including 660 subnational locations, 1990-2023: a systematic analysis for the global burden of disease study 2023. Lancet. (2025) 406(10513):1873–922. 10.1016/s0140-6736(25)01637-x 41092926 PMC12535840

[2] Dell'Isola A Recenti F Giardulli B Lawford BJ Kiadaliri A. Osteoarthritis year in review 2025: epidemiology and therapy. Osteoarthritis Cartilage. (2025) 33(11):1300–6. 10.1016/j.joca.2025.08.015 40914550

[3] Tang S Zhang C Oo WM Fu K Risberg MA Bierma-Zeinstra SM. Osteoarthritis. Nat Rev Dis Primers. (2025) 11(1):10. 10.1038/s41572-025-00594-6 39948092

[4] Harkey MS Gatti AA Jansen MP White MS Tolzman J Parmar A. Osteoarthritis year in review 2025: imaging. Osteoarthritis Cartilage. (2026) 34(1):28–38. 10.1016/j.joca.2025.11.004 41241372 PMC12712793

[5] Prieto-Alhambra D Judge A Javaid MK Cooper C Diez-Perez A Arden NK. Incidence and risk factors for clinically diagnosed knee, hip and hand osteoarthritis: influences of age, gender and osteoarthritis affecting other joints. Ann Rheum Dis. (2014) 73(9):1659–64. 10.1136/annrheumdis-2013-203355 23744977 PMC3875433

[6] Ou J Zhang J Alswadeh M Zhu Z Tang J Sang H. Advancing osteoarthritis research: the role of AI in clinical, imaging and omics fields. Bone Res. (2025) 13(1):48. 10.1038/s41413-025-00423-2 40263261 PMC12015311

[7] Cigdem O Deniz CM. Artificial intelligence in knee osteoarthritis: a comprehensive review for 2022. Osteoarthr Imaging. (2023) 3(3):100161. 10.1016/j.ostima.2023.100161 38948116 PMC11213283

[8] Oettl FC Zsidai B Oeding JF Hirschmann MT Feldt R Fendrich D. Artificial intelligence-assisted analysis of musculoskeletal imaging-a narrative review of the current state of machine learning models. Knee Surg Sports Traumatol Arthrosc. (2025) 33(8):3032–8. 10.1002/ksa.12702 40450562 PMC12310083

[9] Jarraya M Guermazi A Roemer FW. Osteoarthritis year in review 2023: imaging. Osteoarthritis Cartilage. (2024) 32(1):18–27. 10.1016/j.joca.2023.10.005 37879600

[10] Huang YJ Lin CH Miao S Zheng K Lu L Lu Y. Radiographic bone texture analysis using deep learning models for early rheumatoid arthritis diagnosis. J Imaging Inform Med. (2025) 39(3):1289–98. 10.1007/s10278-025-01579-3 40624389 PMC13103098

[11] Moradmand H Ren L. Multistage deep learning methods for automating radiographic sharp score prediction in rheumatoid arthritis. Sci Rep. (2025) 15(1):3391. 10.1038/s41598-025-86073-0 39870749 PMC11772782

[12] Peng Y Huang X Gan M Zhang K Chen Y. Radiograph-based rheumatoid arthritis diagnosis via convolutional neural network. BMC Med Imaging. (2024) 24(1):180. 10.1186/s12880-024-01362-w 39039460 PMC11265088

[13] Cai K Dou D Deng G Zhan Y Huang H Feng Z. Deep learning classification of rheumatoid arthritis in hand radiographs interpretability insights and web application. Immunotargets Ther. (2025) 14:1333–45. 10.2147/itt.S547159 41287803 PMC12640576

[14] Üreten K Maraş HH. Automated classification of rheumatoid arthritis, osteoarthritis, and normal hand radiographs with deep learning methods. J Digit Imaging. (2022) 35(2):193–9. 10.1007/s10278-021-00564-w PMC8921395 35018539

[15] Ma Y Pan I Kim SY Wieschhoff GG Andriole KP Mandell JC. Deep learning discrimination of rheumatoid arthritis from osteoarthritis on hand radiography. Skeletal Radiol. (2024) 53(2):377–83. 10.1007/s00256-023-04408-2 37530866

[16] Caratsch L Lechtenboehmer C Caorsi M Oung K Zanchi F Aleman Y. Detection and grading of radiographic hand osteoarthritis using an automated machine learning platform. ACR Open Rheumatol. (2024) 6(6):388–95. 10.1002/acr2.11665 38576187 PMC11168904

[17] He K Zhang X Ren S Sun J. Deep residual learning for image recognition. In: 2016 IEEE Conference on Computer Vision and Pattern Recognition (CVPR) (2016), 770–8

[18] Huang G Liu Z Van Der Maaten L Weinberger KQ. Densely connected convolutional networks. In: 2017 IEEE Conference on Computer Vision and Pattern Recognition (CVPR) (2017), 2261–9

[19] Dosovitskiy A Beyer L Kolesnikov A Weissenborn D Zhai X Unterthiner T. An image is worth 16×16 words: transformers for image recognition at scale. In: Proceedings of the International Conference on Learning Representations (ICLR) (2021).

[20] Gitto S Serpi F Albano D Risoleo G Fusco S Messina C. AI applications in musculoskeletal imaging: a narrative review. Eur Radiol Exp. (2024) 8(1):22. 10.1186/s41747-024-00422-8 38355767 PMC10866817

[21] Joseph GB McCulloch CE Sohn JH Pedoia V Majumdar S Link TM. AI MSK clinical applications: cartilage and osteoarthritis. Skeletal Radiol. (2022) 51(2):331–43. 10.1007/s00256-021-03909-2 34735607 PMC13197133

[22] Demehri S Kasaeian A Roemer FW Guermazi A. Osteoarthritis year in review 2022: imaging. Osteoarthritis Cartilage. (2023) 31(8):1003–11. 10.1016/j.joca.2023.03.005 36924919 PMC12224773

[23] Hayashi D Roemer FW Guermazi A. Osteoarthritis year in review 2024: imaging. Osteoarthritis Cartilage. (2025) 33(1):88–93. 10.1016/j.joca.2024.10.009 39490728

[24] Binvignat M Pedoia V Butte AJ Louati K Klatzmann D Berenbaum F. Use of machine learning in osteoarthritis research: a systematic literature review. RMD Open. (2022) 8(1):e001998. 10.1136/rmdopen-2021-001998 35296530 PMC8928401

[25] Perronne L Binvignat M Foulquier N Saraux A Laredo JD De Margerie-Mellon C. Algorithmic approaches in hand imaging for rheumatic musculoskeletal diseases: a systematic literature review. Semin Arthritis Rheum. (2025) 73:152750. 10.1016/j.semarthrit.2025.152750 40349420

[26] Singla S Eslami M Pollack B Wallace S Batmanghelich K. Explaining the black-box smoothly-a counterfactual approach. Med Image Anal. (2023) 84:102721. 10.1016/j.media.2022.102721 36571975 PMC9835100

[27] Zhang C Peng J Wang L Wang Y Chen W Sun M- A deep learning-powered diagnostic model for acute pancreatitis. BMC Med Imaging. (2024) 24(1):154. 10.1186/s12880-024-01339-9 38902660 PMC11188273

[28] Borys K Schmitt YA Nauta M Seifert C Krämer N Friedrich CM. Explainable AI in medical imaging: an overview for clinical practitioners - saliency-based XAI approaches. Eur J Radiol. (2023) 162:110787. 10.1016/j.ejrad.2023.110787 37001254

[29] Ahmed F Naz NS Khan S Rehman AU Ismael WM Khan MA. Explainable artificial intelligence (XAI) in medical imaging: a systematic review of techniques, applications, and challenges. BMC Med Imaging. (2026) 26(1):37. 10.1186/s12880-025-02118-w 41491470 PMC12809972

[30] Mohammadi S Salehi MA Jahanshahi A Shahrabi Farahani M Zakavi SS Behrouzieh S. Artificial intelligence in osteoarthritis detection: a systematic review and meta-analysis. Osteoarthritis Cartilage. (2024) 32(3):241–53. 10.1016/j.joca.2023.09.011 37863421

[31] Muhammad D Bendechache M. Unveiling the black box: a systematic review of explainable artificial intelligence in medical image analysis. Comput Struct Biotechnol J. (2024) 24:542–60. 10.1016/j.csbj.2024.08.005 39252818 PMC11382209

